# The impact of medical insurance policies on the hospitalization services utilization of people with schizophrenia: A case study in Changsha, China

**DOI:** 10.12669/pjms.293.3273

**Published:** 2013

**Authors:** Yi Feng, Xianjun Xiong, Qiuji Xue, Lan Yao, Fei Luo, Li Xiang

**Affiliations:** 1 Yi Feng, School of Medicine and Health Management, Tongji Medical College, Huazhong University of Science and Technology, Wuhan, China.; 2 Xianjun Xiong, China Health Insurance Research Association, Beijing, China.; 3 Qiuji Xue, School of Medicine and Health Management, Tongji Medical College, Huazhong University of Science and Technology, Wuhan, China.; 4 Lan Yao, School of Medicine and Health Management, Tongji Medical College, Huazhong University of Science and Technology, Wuhan, China.; 5 Fei Luo, School of Medicine and Health Management, Tongji Medical College, Huazhong University of Science and Technology, Wuhan, China.; 6 Li Xiang, School of Medicine and Health Management, Tongji Medical College, Huazhong University of Science and Technology, Wuhan, China.

**Keywords:** Medical insurance policy, Schizophrenia, Hospitalization service utilization, Impact

## Abstract

***Objective:*** To evaluate the impact of two medical insurers’ policies on the hospitalization of people with schizophrenia and the economic burden they faced during a period of rapid health services reform in China.

***Methodology:*** A comparative analysis was made of Urban Employee-Basic Medical Insurance (UE-BMI) and Urban Residents-Basic Medical Insurance (UR-BMI) policies on the medical management of schizophrenics, and was compared with hospitalization expenses, insurer reimbursement data and other information collected from the HMO (health maintenance organization) and social insurance agencies on the care of people with schizophrenia in Changsha in 2010. In-depth interviews were also conducted with relevant managers.

***Results:*** Compared with inpatients covered by UR-BMI, the inpatients of UE-BMI were admitted to higher level medical institutions and were prescribed expensive second generation antipsychotics (SGA) medicines. Nevertheless, the hospitalization service utilization and cost of inpatients’ hospitalization under UE-BMI were far less than that of inpatients under UR-BMI.

***Conclusions:*** The insurance level difference between two medical insurance schemes influences the treatment regimens and benefits received by patients. Furthermore, the integration of schizophrenia management into the outpatient services pooling fund for special diseases(OS-PFSD) can appropriately reduce hospitalization utilization, which, together with the payment way reform and the prescription of reasonable medications, can significantly reduce the overall hospitalization cost for patients.

## INTRODUCTION

The incidence of mental illness in China has increased in recent years concurrent with and potentially linked to the heightened competitive pressures stemming from the nation’s rapid economic development and social transition. The Ministry of Health reported in 2007 that the incidence of mental illness was 15% with severe mental illness prevalence at 1%. These figures placed mental illness as the greatest contributor to China’s total burden of disease, generating 20% of that burden, surpassing tumors and cardiovascular diseases. Mental illness has become China’s major public health problem, bringing with it significant social consequences. Schizophrenia ranks as the most prevalent serious mental illnesses with a prevalence rate of about 1%. Current estimates are that there are about eight million people in China suffering from schizophrenia. This number is increasing year by year.^1^ At a personal level, people with serious mental illness are frequently unable to work or live normally, they have limited opportunities to earn a living and often become poor. Family breakdown, suicide or other tragic consequences may ensue.

At this still experimental stage of the rapid development of medical insurance in China, there is likely to be great variation in medical insurance policies’ coverage of a disease between cities, or between the coverage provided by different medical insurance systems in one city. This raises the question as to how medical insurance policy differences and institutional arrangements impact on service utilization by people requiring treatment for schizophrenia. To date there has been only limited research on this topic in China and it has been peripherally addressed in some other recent researches. For example, Cai et al2 discovered that schizophrenics with medical insurance have a higher treatment rate than those without medical insurance. Bu et al3 found that patients who receive higher insurance benefits offered by UE-BMI have higher treatment rate than those with UR-BMI who receive a lower reimbursement. Guo et al,4 Yang et al5 and Xie6 all discovered through studies that patients covered by medical insurance utilized more SGA than those with no medical insurance. Each of these findings provided important background for this research.

This study aimed to analyze the impact of different medical insurance policies in one region on the hospitalization services utilization of patients diagnosed with schizophrenia and their consequent economic burden. Furthermore, it evaluated the implementation of the health reforms.

## METHODOLOGY

***Materials: ***A field survey was conducted to collect the policy documents and implementation methods for the medical insurance management of schizophrenia in both UE-BMI and UR-BMI at the Changsha municipal HMO. In addition, data on hospitalization expenses and reimbursement levels was collected along with anonymized data on claims for hospitalization for schizophrenia from the 2010 database of the social insurance agencies by retrospective data survey.

***Interview: ***The data analysis was supplemented with in-depth interviews conducted with fifteen managers of Changsha Municipal HMO, Social Insurance Agencies, Health Bureau, three specialized mental health hospitals and five community health service centers. These were conducted in order to reveal the practical implications of the medical insurance management policies, their impact on, and the problems faced by people with schizophrenia in the utilization of outpatient and inpatient medical services in Changsha. Information was obtained from the interviewees using semi-structured interviews that allowed new questions to be brought up during the interviews, depending upon what the interviewees say in their answer.^7^

***Data analysis: ***An analysis comparing the two medical insurance management policies for people with schizophrenia was carried out. It covered hospitalization rates, lengths of stay, inpatient flow, hospitalization expense levels and allocation, hospital medication and utilization of other hospitalization services in Changsha in 2010. Data were analyzed using SPSS for Windows (Version 16.0).

## RESULTS

Changsha is the provincial capital of Hunan Province, China. It has a population of 6.51 million. As is the case in most other Chinese cities, the principal urban medical insurance programs in Changsha City are UE-BMI and UR-BMI. In 2010, these schemes raised funds from government and individuals’ subscriptions amounting to 1,930.01 million RMB for the UE-BMI and 114.08 million RMB for the UR-BMI. The schemes each covered 1.15 million people (UE-BMI) and 1.03 million people (UR-BMI).

***Comparison of medical insurance management of schizophrenia by the two medical insurance systems of Changsha:***

A systematic analysis of the policy documents revealed three main differences. First, UE-BMI integrated schizophrenia management into the outpatient services pooling fund for special diseases (OS-PFSD) while UR-BMI made no provision for financially supporting outpatient care of these patients. Secondly, UE-BMI provides higher reimbursement for hospitalization than UR-BMI. Thirdly, While UR-BMI maintains the traditional fee-for-service method of calculating reimbursements for both outpatient and hospitalization, UE-BMI has introduced some new reimbursement methods. For example, it has moved from patient reimbursement to capitation payment to designated providers for outpatient services. These providers are paid a standard 80 RMB per person per month from the risk-pooling fund for providing outpatient services. No further patient contribution is to be required. UE-BMI still reimburses members on a fee-for-service basis for acute inpatient admissions. For long-term inpatients with specified serious conditions, it pays “fee for unit according to hospital level”. Specifically, designated tertiary-level, secondary-level and first-level medical institutions are required to be completely responsible for providing hospitalization for standardized payments of “120 RMB/day, 110 RMB/day and 70 RMB/day” respectively. This is paid by the risk-pooling fund without any individual out-of-pocket contribution.

***Comparison of the general conditions for schizophrenia inpatient management between two medical insurance schemes in Changsha:***

Table-I illustrates the findings of the analysis. The experience of patients hospitalized with schizophrenia was significantly influenced by the insurance scheme that covered their illness. Patients with UR-BMI coverage experienced three times longer average length of stay and the insurer made six times higher contributions for inpatient care compared with those made by UE-BMI.

Inpatients covered by UE-BMI principally received treatment from tertiary medical institutions (81.4%) while inpatients covered by UR-BMI principally received treatment from secondary medical institutions (73.3%). However, very few patients received inpatient care in first-level medical institutions, with no inpatients covered by UR-BMI receiving this form of care.

The cost comparison reveals that despite its much higher reimbursement rates, UE-BMI outlaid half the amount spent by UR-BMI per patient admitted during the year. This directly relates to the far longer average length of stay per capita of UR-BMI covered patients.

***Comparison of the cost of antipsychotics dispensed to inpatients covered by two medical insurance schemes in Changsha:***

As the results in Table-II show, antipsychotic drugs costing under 10 RMB constituted the most categories and were the most utilized by inpatients of both medical insurances, making up over 87% of the total drug use. Hence, prescribed antipsychotics were generally cheap and the vast majority of patients used cheap drugs. However, comparing the two insurers, the proportion of expensive drugs prescribed for UE-BMI patients was higher than for those under UR-BMI. This is also reflected in the cost proportion.

***Prescribing patterns and consequent costs of FGA and SGA:***

**(1)** The use as well as consequent cost of FGA and SGA in the treatment of inpatients covered by the two medical insurance schemes.

Table-III illustrates significant differences between the two insurance schemes with respect to inpatients being prescribed first and second generation antipsychotics. Those with UE-BMI coverage were rarely prescribed FGA alone (3%) and most inpatients received SGA alone (58%). Inpatients covered by UR-BMI faced the opposite situation with most inpatients receiving FGA alone (42.5%) and the proportion receiving SGA alone (32.8%) was far less than UE-BMI inpatients. Furthermore, regardless of the medication prescribed, inpatients of UR-BMI almost attained a higher per capita cost for antipsychotics, total drugs and hospitalization than the UE-BMI inpatients.

***(2) Expense characteristics of using different medication by inpatients.***

For both medical insurances inpatient, prescription of FGA alone was the lowest antipsychotics cost per capita. FGA alone was the prescription of choice for 98% of UR-BMI inpatients. Despite this, that cohort’s total drugs cost per capita was the highest among three kinds of medications. Comparing FGA prescription alone and SGA prescription alone for both medical insurers inpatients, antipsychotics cost per capita for SGA prescription alone constitutes between 23-50% of the total drug cost during the admission while FGA prescription alone constitutes only 2% of the total drug cost. The antipsychotics cost per capita for SGA prescription alone was between 8 and 70 times higher than that for FGA prescription alone. These data illustrate the well documented evidence that while the prescription of FGA alone produces lower antipsychotics cost per capita, it risks introducing Extrapyramidal motor side-effect (EPS) and requires higher expenses for anti-EPS drugs for their control,^8^^-^^11^ significantly increasing the total drugs cost. On the other hand, the utilization of SGA alone produces higher antipsychotics cost per capita, but minimizes side-effects and needs lower expenses for anti-EPS drugs, thus decreasing the total drugs cost. In addition, for both medical insurances’ inpatients, SGA prescriptions alone were accompanied by lower diagnostic tests cost per capita. Finally, 98% of inpatients receiving FGA alone had longer length of stay per capita and consequently higher hospitalization cost than those utilizing SGA alone.

Taken together, the data indicate that the prescription of SGA is beneficial for reducing total drugs cost and diagnostic tests cost as well as shortening length of stay, thus reducing the hospitalization cost.

## DISCUSSION

***The impact of insurance level differences between two medical insurances on the hospitalization service utilization of patients:***

The data analysis stated that: firstly, the inpatients of UE-BMI utilized higher level medical institutions than inpatients of UR-BMI. Secondly, the inpatients of UE-BMI had higher proportion of patients receiving expensive drugs than those with UR-BMI coverage. Thirdly, the SGA prescription rate of inpatients of UE-BMI was significantly higher than those of UR-BMI.

The beneficiaries of UE-BMI receive higher levels of reimbursement than those with UR-BMI coverage. The statistical yearbook of Changsha reports that the annual average wage of urban employees in 2010 was 40,157 RMB. This is significantly higher than the per capita disposable income of urban residents (22,814 RMB) that year.^12^ Mr. Ou, the director of Changsha Mental Hospital, advised that “many schizophrenic patients of UR-BMI have neither a stable job nor an income source, leaving them in a poor financial situation. The UR-BMI’s provision of a lower reimbursement level restricts their utilization of health services to a large extent.” The analysis undertaken in this research indicates that differences in reimbursement level and income level are significant causative factors of abovementioned situation. Most of cities in China have different insurance levels in different medical insurance schemes, due to the different establishment time. Those policies and subsequent practices directly influence the treatment regimens and benefits received by patients and have a substantial impact on accessibility and equity of health services.

**Table-I:** Comparison of the general conditions for schizophrenia inpatient management between two medical insurance schemes in Changsha

	*UE-BMI*	*UR-BMI*	*Total*
Hospitalizations	70	457	527
First-level	4	0	4
Secondary-level	9	335	344
Tertiary-level	57	122	179
Number of inpatients	66	423	489
Annual hospitalizations per capita (times)	1.1	1.1	1.1
Annual length of stay per capita (day)	50.6	187.1	164.3
Average cost per hospitalization (RMB)	8613.9	23115.3	21189.2
Average cost paid by risk-pooling fund per hospitalization (RMB)	5977.6	10378.7	9794.1
Average practical reimbursement rate per hospitalization (%)	69.4	44.9	46.2
Annual average hospitalization cost per capita (RMB)	9135.9	24973.3	22835.8
Annual cost paid by risk-pooling fund per capita (RMB)	6339.9	11212.9	10555.2
Annual practical reimbursement rate per capita (%)	69.4	44.9	46.2

**Table-II:** Comparison of the cost of antipsychotics dispensed to inpatients covered by two medical insurance schemes in Changsha in 2010

	*Price-* *range* *(per bottle)*	*Drug categories*	*Number of use* *s*	*Proportion * *of * *total users (%)*	*Cost proportion* *(%)*
UE-BMI					
	< 10	9	2400	87.1	24.6
	10～20	3	96	3.5	5.4
	20～50	4	190	6.9	13.0
	50～100	4	20	0.7	5.5
	>100	4	50	1.8	51.5
UR-BMI					
	<10	11	101828	98.6	61.1
	10～20	5	364	0.4	8.0
	20～50	5	1008	0.9	17.0
	50～100	4	45	0.04	3.6
	>100	5	66	0.06	10.3

**Table-III:** The use as well as consequent cost of FGA and SGA in the treatment of inpatients covered by the two medical insurance schemes

	*Utilization of antipsychotics*	*Users (person)*	*Ratio (%)*	*Antipsychotics cost per capita (RMB)*	*Total drugs cost per capita (RMB)*	*D* *iagnostic * *tests cost per capita (RMB)*	*Hospitalization cost per capita (RMB)*	*Length of stay per capita (day)*
UE-BMI	FGA alone	2	3	13.2	661.7	2915.3	6114.8	29
	SGA alone	36	58	921.5	1834.9	2733.4	8834.1	45
	Both FGA and SGA	24	39	734.6	2226.3	3796.1	10329	53
UR-BMI	FGA alone	172	42.5	115.2	5516.6	2284.9	35358.8	297
	SGA alone	133	32.8	865.7	3841.1	2623.8	19535.2	153
	Both FGA and SGA	100	24.7	1307.3	3168.9	2868.2	13972.6	71

**Note:** The antipsychotics cost per capita in this table only relate to FGA or SGA costs (excluding adjuvant drug), with the total drugs cost per capita including the cost of all drugs used by the patients during the hospitalization.

***The impact of integrating*** ***schizophrenia management into OS-PFSD on patients’ utilization of hospitalization and hospitalization cost:***

Mr. Dai, Deputy Director General of Changsha Municipal Medical Insurance Bureau, stated that “since UE-BMI’s integration of schizophrenia management into OS-PFSD, less ill patients and those recovering patients who can be treated by both outpatient service and hospitalization were transferred to outpatient treatment, thus decreasing the inpatients amount and length of stay. Contrarily, UR-BMI has not yet to implement such reform. Therefore, to get reimbursement for their care, some patients who could be treated successfully as outpatients choose hospitalization treatment and recovering patients tended to prolong their length of stay. ” Furthermore, the hospitalization cost will surely be decreased by decreasing the length of stay. Yu et al^13^ proposed that the establishment of a pooling fund of outpatient services is beneficial for early disease detection, early diagnosis and early treatment, which can encourage appropriate use of health services to the maximum extent and avoid delays in treatment that lead to minor illness unnecessarily becoming serious illness with its associated higher costs of care. This research supports the policy for the integration of schizophrenia management into OS-PFSD for its positive impact on controlling cost as well as guiding patients to reasonable treatment.

***The impact of payment way reform on the hospitalization cost:***

Changsha UE-BMI implemented the payment way of “fee for unit according to hospitals’ level” for long-term inpatients. We were informed during the interview that the reform of such payment way not only strictly controls the unit fee of long-term inpatient of UE-BMI and decreases the unnecessary expenses in unit time, but also controls the suppliers’ behavior of providing long-term hospitalization, thus controlling the hospitalization cost. Contrarily, the UR-BMI still implemented the payment way of fee-for-service-items for all inpatients, and patients of UR-BMI are unable to be reimbursed through outpatient service, thus making both supply and demand parties tend to prolong length of stay and finally increasing the hospitalization cost. The comparison demonstrated that the payment way reform of Changsha UE-BMI achieved good effect although it is not perfect and only beneficial for part of patients.

***Limitation of the study: ***We only collected the hospitalization cost levels and insurance reimbursement data of schizophrenics in 2010. Data from the period prior to the reform could not be obtained hence no vertical comparison is being made of the management and cost of schizophrenia inpatients of the two medical insurance schemes before and after reform. It is still a limitation of this study although corresponding addition was made through the interview of managers. In addition, no patients or their relatives were involved in the interviews. Their opinions and thoughts could have been useful in getting more insight in the matter.

## CONCLUSION

The insurance level difference between two medical insurance schemes influences the treatment regimens and benefits received by patients. Furthermore, the integration of schizophrenia management into OS-PFSD can appropriately reduce hospitalization utilization, which, together with the payment way reform and the prescription of reasonable medications, can significantly reduce the overall hospitalization cost for patients. It is an important reason for UE-BMI inpatients to incur significant low hospitalization cost than those with UR-BMI coverage.

## Authors' contributions

YF conducted the data analysis, drafted the manuscript and contributed to subsequent revisions. LX and LY conceived the idea for the study, participated in study design, and contributed to the data analysis and to the drafting and revising of the manuscript. Other authors contributed to implementing the study, analyzing the data, and editing of the final manuscript. All authors read and approved the final manuscript.
